# Role of multi-detector computed tomography (MDCT) in management of post percutaneous nephrolithotomy (PCNL) bleeding

**DOI:** 10.12688/f1000research.2-253.v1

**Published:** 2013-11-22

**Authors:** Arvind P Ganpule, Darshan H Shah, Sanika A Ganpule, Ravindra B Sabnis, Mohan M Rajapurkar, Mahesh R Desai

**Affiliations:** 1 Department of Urology, Muljibhai Patel Urological Hospital, Nadiad, India; 2 Department of Radiology, Muljibhai Patel Urological Hospital, Nadiad, India; 3 Department of Nephrology, Muljibhai Patel Urological Hospital, Nadiad, India

## Abstract

**Objective: **To evaluate the role of multidetector computerized tomography (MDCT) angiography in post percutaneous nephrolithotomy (PCNL) bleed and compare findings with conventional angiography (CA).

**Material and methods: **We conducted a retrospective analysis of patients who had post PCNL bleeding and subsequently underwent a MDCT angiography followed by CA. We reviewed eight patients, who presented between January 2009 and January 2013. We performed a MDCT angiography on a 16 slice GE bright speed CT scanner. All angiographies were done by using the Digital Subtraction Angiography Suite. The angioembolisation, if required, was conducted by an interventional nephrologist, specializing in therapeutic embolisation.

**Results:** The mean age of the patients was 42±17 years. Mean time of post PCNL bleed presentation was 10.06±7.9 days. Five patients presented with aneurysm and three presented with an AV fistula with pseudoaneurysm. The right renal unit was involved in six cases and the left in two cases. The lower polar segmental artery was involved in six cases and the upper polar artery in two cases. The CA and MDCT findings matched in all cases and the MDCT helped the clinician to assess and embolise the appropriate arterial tree.

**Conclusion: **MDCT is rapid, reproducible and noninvasive. MDCT angiography performed in the setting of post PCNL bleeding provides an accurate assessment of the site and nature of bleeding. The MDCT angiography matched the CA findings in all patients in the present study.

## Introduction

Bleeding after percutaneous nephrolithotomy (PCNL) occurs in 0.3 to 1% of patients ^1^. The majority of episodes are self limiting and do not require intervention. Life threatening bleeding following percutaneous renal surgery requires accurate imaging, which helps the treating physician to make a clinical decision regarding management. Various non-invasive investigative modalities such as "grayscale" ultrasonography and Doppler ultrasound have previously been key in the decision-making process. Conventional angiography (CA) can be used in both the diagnosis and treatment of bleeding following PCNL. However, CA has limitations and pitfalls including its inability to accurately identify bleeding. In addition, although there is a low incidence of access site complications, CA requires expertise when used as an intervention ^2^.

Multidetector computerisd tomography (MDCT) is currently being used prior to endourologic intervention, and its role in managing bleeding in the gastrointestinal tract has been assessed in the past ^3, 4^. There is a paucity of literature describing its utility in post PCNL bleeding. A MDCT would potentially help in assessing the vascular anatomy, the site of bleeding and hence subsequently aid intervention. In this article, we assess the utility and accuracy of findings of multidetector computerized tomography (MDCT) angiography in patients with post PCNL bleeding. The findings were compared with those seen using CA.

## Material and methods

A retrospective chart review of all patients who presented with post PCNL bleeding was conducted. All patients were admitted prior to the procedure. Among these patients six patients had a PCNL done at our hospital while two were referred from another hospital. Informed written consent regarding the procedure and future use of this data in any form in any meeting or publication was obtained from each patient included in the analysis. Those patients evaluated with MDCT angiography and who subsequently underwent CA and embolisation between January 2009 and January 2013 were included in the analysis. MDCT angiography was conducted on a 16 slice GE Bright Speed ^TM^ CT (GE Healthcare, US) scanner. The findings for MDCT were compared with those with CA. All MDCT angiographies were performed and reported by a single radiologist. All the CAs were performed by an interventional nephrologist.

### Technique of MDCT angiography

No oral or bowel contrast was administered during MDCT angiographies. The contrast agent iopromide (Ultravist), 1.5–2ml/kg body weight was injected in an adequate sized forearm vein using a pressure injector. A triphasic acquisition of the images was performed. The scan ranged from the diaphragm to the inferior pubic ramus. An unenhanced MDCT was obtained at baseline. The locator was positioned over the descending aorta and images were acquired once the contrast was detected in the descending aorta. This constituted the arterial phase. At an interval of 40–60 seconds after contrast injection, the second scan was performed to obtain the images in the venous phase. The delayed scan was acquired after 10 minutes for excretory images. The section thickness was kept at 1mm, the reconstruction thickness was kept at 0.8mm.The gantry rotation time of 0.5 seconds was kept for all the three phases.

The data was processed with the Maximum intensity projection (MIP) and Volume rendering (VR) techniques. These are methods for 3D reconstruction and image display. Both are post processing techniques with their own advantages and disadvantages ^5^. The parameters noted included the type, size and site of the lesion, the method of embolisation and the conventional and MDCT angiography findings (success being defined as complete arrest of bleeding). The radiologist while reporting the CT scan noted the following parameters:

1) Presence of arteriovenous fistula, pseudoaneurysm or aneurysm.

2) Presence of any residual stones.

3) Presence of perinephric sub capsular hematomas.

4) Presence or absence of clots in the collecting system.

5) Size of the lesion and its relation to primary, secondary or quaternary feeders.

All angiographies were done on Digital Subtraction Angiography Suite (Medico India Ltd). Prior to embolisation a flush angiogram was performed, following which a selective angiogram was obtained. The angioembolisation, if required, was performed by an interventional nephrologist, specialised in therapeutic embolisation. The vascular system was accessed through the ipsilateral common iliac artery; if access was not possible through a femoral route access was gained through an axillary route. The decision to use a coil or a gel foam stick for embolisation was taken depending on the lesion and the diameter of the lesion to be embolised.

## Results

The mean age of the patients was 42±17 years. There were six males and two females. Mean time of post PCNL bleed presentation was 10.06±7.9 days. Five patients presented with aneurysm and three presented with an arteriovenous (AV) fistula with pseudoaneurysm. The right renal artery was involved in six cases and the left in two cases. The lower polar segmental artery was involved in six cases and the upper polar artery in two cases. The CA and MDCT findings matched in all cases. In all cases, MDCT helped the clinician to assess and embolise the appropriate arterial tree. Details of each case are summarized in Table 1.

**Table 1.** Summary of clinical findings of patients with PCNL bleeds using MDCT and CA.

Case	Age	Procedure done	Day of presentation	MDCT angiography finding	Conventional angiography finding	Associated findings
**1**	28	Standard PCNL	7	Aneursymal dilatation of lower pole segmental artery of right kidney	Aneursymal dilatation of lower pole segmental artery of right kidney	Clots in PCS No residual stones
**2**	60	Standard PCNL	6	Large pseudo-aneurysm of 32mm, supplied by terminal branch of posterior segmental artery	Large pseudo-aneurysm supplied by terminal branch of posterior segmental artery	Clots in PCS and Residual stones Perinephric hematoma
**3**	46	Standard PCNL	8	AV fistula of post segmental artery at upper pole of left kidney, 10mm in size	Left upper pole AVF from Posterior segmental branch	Clots in PCS and ureter No residual stone
**4**	13	Standard PCNL	5	Persistent leakage of contrast from upper pole vessel, suggestive of AV fistula	Persistent leakage of contrast from upper pole vessel, suggestive of AV Fistula	Clots in PCS No residual stone
**5**	63	Standard PCNL	15	Right renal lower polar segmental artery aneurysm of 12mm	Right renal lower polar segmental artery aneurysm	Right perinepheric space hematoma measuring 112 × 89 × 88mm No residual stone Clots in PCS
**6**	54	Miniperc	4	Right renal lower pole AV fistula with pseudo aneurysm about 1.4cm × 0.9cm × 0.9cm	Right renal lower pole pseudo aneurysm with AV fistula	Clots within the right renal pelvicalyceal system and right ureter No residual stone
**7**	30	Standard PCNL	12	Aneurysmal dilatation of 7.7 × 6.6mm, of lower pole segmental branch of right upper renal artery	Aneurysm of lower pole segmental branch of right upper renal artery	Extravasation of contrast into perinephric collection of 15 × 8cm ^2^
**8**	42	Standard PCNL	28	Aneurysm of left lower pole segmental artery 5 × 6mm	Left renal lower pole arterial aneurysm	No residual stone

MDCT: Multidetector computerized tomography, PCNL: Post percutaneous nephrolithotomy, PCS: Pelvicalyceal system.

### Case 1

**Figure 1.**
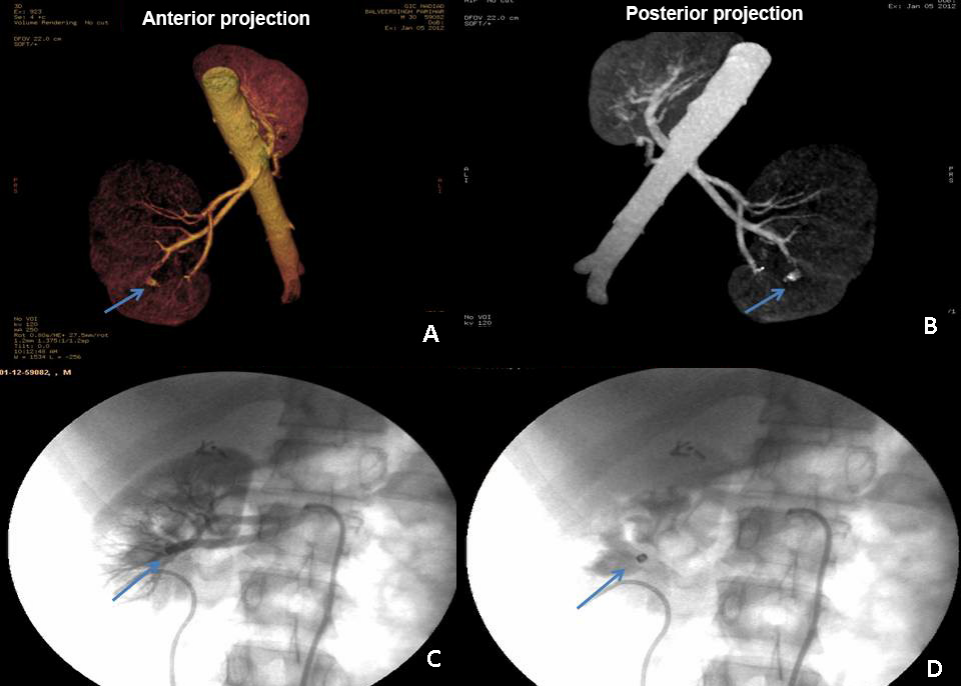
Case 1. **a**) Aneurysmal dilatation seen on MDCT in a branch of the posterior segmental artery (anterior projection; arrow). **b**) The same lesion seen on posterior projection (arrow). **c and d**) A platinum coil used for embolisation (arrows).

A 28 year old male presented with gross hematuria with clots, a hemoglobin drop of 3gm/dl following 7 days of PCNL, and required two units of blood transfusion. Upon MDCT evaluation he was found to have right lower pole segmental artery aneurysm. Further it also showed clots in pelvi calyceal system (PCS). There were no residual calculi and hematoma. These findings matched with findings on CA. A coil embolisation was performed, following which the hematuria settled ( Figure 1).

### Case 2

**Figure 2.**
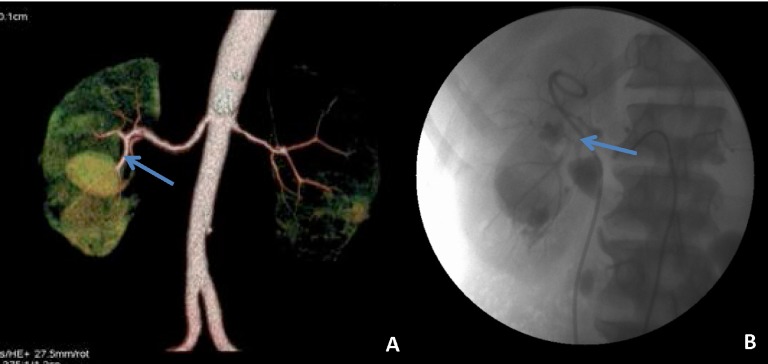
Case 2. **a**) A MDCT angiogram showing a large pseudoaneursym from the posterior segmental artery (anteroposterior projection) The arrow shows the feeding vessel. **b**) A conventional angiogram showing a pseudoaneursym from the branch of the posterior segmental (lateral projection). The arrow shows the feeding vessel.

A 60 year old male presented with gross hematuria with a need for blood transfusion following 6 days of PCNL in a solitary functioning right kidney. A Doppler ultrasound revealed the possibility of an aneurysm. Prior to CA, a MDCT angiography revealed the presence of the aneurysm. Considering the fact that the size of the lesion was more than 1cm and the lesion had multiple feeders, the chance of successful embolisation was guarded. However in view of the solitary kidney status, an attempt of super selective embolisation was done. CA showed AV fistula with pseudoaneurysm, supplied by posterior segmental artery ( Figure 2a). The MDCT angiography findings helped the clinician to assess the exact size, location and number of feeding arteries that could be embolised. In addition, the MDCT also revealed the presence of residual stones and the presence of a perinephric hematoma. After diagnostic CA which confirmed findings ( Figure 2b) of MDCT embolisation was done.

### Case 3

**Figure 3.**
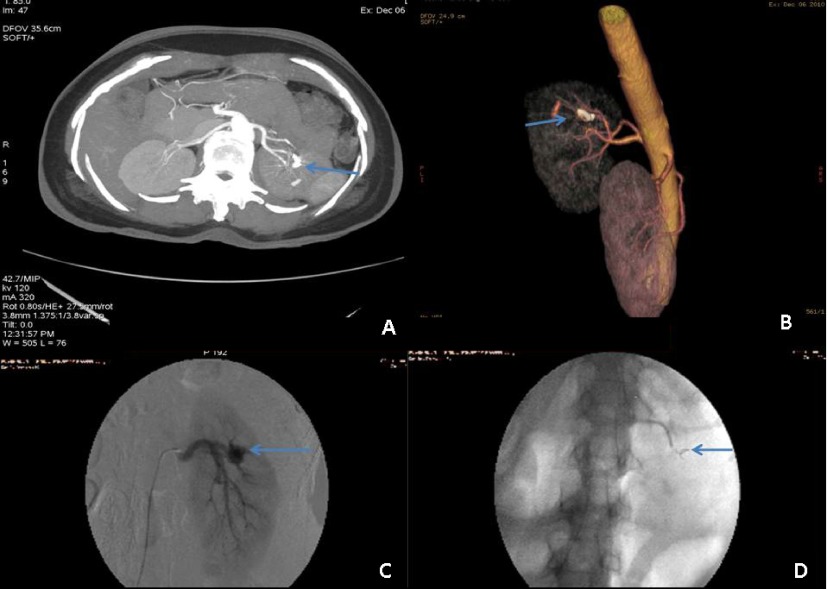
Case 3. **a and b**) Axial cuts and lateral MIP images showing AV fistula in a branch of the posterior segmental artery. Arrow shows the location of AV fistula. **c**) Conventional angiography confirms the presence of AV malformation in the branch of the posterior segmental. Arrow shows the location of AV fistula on CA. **d**). Successful embolisation. Arrow shows the position of the coil.

A 46 year old female patient underwent left PCNL, and on the 8 ^th^ post operative day developed hematuria. Emergency cystoscopy with clot evacuation with left ureteric and pelvic clots evacuation was done by Fogarty catheter and double J (DJ) stent placed. A therapeutic embolisation with a coil was conducted. MDCT angiography revealed a persistent fistula after embolisation, no residual calculi or hematoma. The MDCT angiogram shows AV fistula of upper polar branch of posterior segmental artery ( Figure 3a and 3b). This helped in accurately embolising the concerned vessel ( Figure 3c and 3d). Hematuria settled after successful embolisation.

### Case 4

A 13 year old male developed hematuria and fever on the 5 ^th^ post operative day following PCNL for a right renal stone. After stabilizing the patient hemodynamically MDCT angiography was done. MDCT showed persistent leakage of contrast from upper pole vessel with clots in PCS and no residual calculi. These findings were confirmed on CA (images not available). Selective angioembolisation of upper pole vessel was done and hematuria settled.

### Case 5

A 63 year old male underwent right PCNL for multiple renal calculi. He had hematuria after 15 days. He needed emergency cystoscopy with clot evacuation and right DJ stenting. A blood transfusion was given. MDCT angiography showed right renal lower segmental artery aneurysm, which was confirmed on CA and embolisation was performed (images not available).

### Case 6

A 54 year old male patient developed hematuria on the 4 ^th^ day following a right "Miniperc" PCNL. MDCT angiography showed right lower pole AV malformation with pseudoaneurysm with clots in PCS. Findings were confirmed on CA and selective angioembolisation was done (images not available).

### Case 7

**Figure 4.**
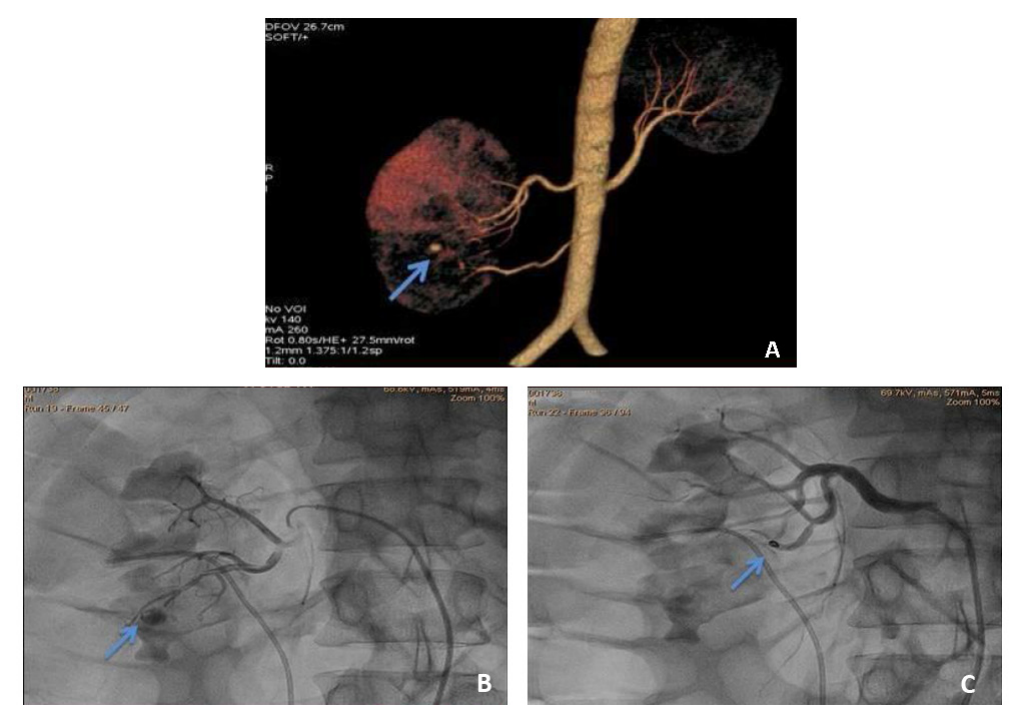
Case 8. **a**) The MDCT angiogram shows the presence of two renal arteries. In addition it also shows aneurysmal dilatation of lower pole segmental branch of right upper renal artery (arrow). **b and c**) A conventional angiogram done the catheter is manipulated into the appropriate branch and embolisation done (arrows).

A 30 year old male patient with no comorbidities underwent right PCNL with DJ stenting for a right renal pelvic stone. On the 12 ^th^ post operative day he developed hematuria with clot retention. Cystoscopy with clot evacuation was done. MDCT angiography showed extravasation of contrast from lower pole calyces into perinephric collection of 15 × 8cm ^2^ size and aneurysm of the lower pole segmental branch ( Figure 4a) of the right upper renal artery. MDCT angiography revealed the presence of two renal arteries, with the AV malformation in the upper artery. He was successfully embolised ( Figure 4b and 4c) by CA after confirmation of MDCT findings.

### Case 8

42 year male, known diabetic underwent left PCNL. Procedure was uneventful. On 28 ^th^ post operative day he had hematuria which increased in amount on the next day. On evaluation found to have left lower pole segmental artery aneurysm on MDCT. CA showed the same findings. He underwent selective angioembolisation following which hematuria settled (Images not available).

## Discussion

Bleeding after PCNL is a stressful situation for both the patient and the operating surgeon. A clinical decision needs to be made depending on the nature of bleeding (arterial or venous), site of bleeding and the type of vascular anomaly. An ideal imaging modality should be minimally invasive and as non-traumatic as possible. It should have a high sensitivity and specificity and should be able to guide the clinician in the management of the patient. There are multiple factors influencing the ability to visualize active bleeding using CT including the type of bleeding, the type of CT machine used and the experience of the radiologist. If the bleeding is exanguinating, angioembolisation is the treatment of choice. Although there are no "benchmark" markers to decide the need for angioembolisation, clinicians decide on the need to embolise depending on the presence of hematuria with clots, hypotension, and/or a serially decreasing hematocrit that does not respond to conservative management ^6^. Richstone and colleagues report in their series the incidence for the need for embolisation to be 1.2% of total PCNL cases ^7^. They concluded that arterial pseudoaneurysms, followed by AV fistulas are the most common findings seen on CA ^7^. The well known limitations of CA are that it reveals the cause of hematuria only in a scenario where there is an active bleed. Second, venous bleeding and bleeding associated with infection may not be readily detected by CA ^8^.

The advantages of MDCT angiography have been well established in gastrointestinal bleeding ^2, 3^. This has led to an increased interest in using the same modality in detecting the cause of bleeding after PCNL. Besides being non invasive and having short scanning times, MDCT provides thinner collimation, greater anatomic coverage, which increases its diagnostic scope ^2, 3^. Movement artifacts from respiration and peristalsis hamper CA; this is eliminated with a rapidly performed MDCT. In addition, MDCT does not require bowel preparation.

MDCT in such a setting helps to determine the presence of residual stones and postoperative collections. In addition, it also allows the interventionist to plan how to target the offending vessel ^3^. Although there are plenty of reports describing the utility of CA and selective angiography in post PCNL bleeding, there have only been anecdotal case reports describing the use of MDCT angiography ^8^. Further, as far as the authors are aware, there have been no reports comparing these two modalities.

In our experience a MDCT angiogram provides a "roadmap" for the intervening clinician. Depending on the size and type of lesion, the intervening physician can plan the intervention, including selection of the embolisation material, the endovascular approach to the offending vessel and can even select the most appropriate method of hemostasis (gelfoam/coil etc). The "roadmap" has the potential to effectively decrease the number of angiographic series, saving time, radiation exposure and the amount of contrast agent used during intervention. This needs to be assessed objectively in further studies. The second advantage of this approach is in the event that the MDCT angiogram is completely negative it decreases the chance of finding a significant bleeding on interventional angiography and may suggest the need for a more conservative approach for managing the bleeding. Studies have shown that AV fistulas smaller than 3–4mm have a good chance of successful angioembolisation in comparison to larger ones ^9^. The exact nature of the problem can be discerned on a MDCT angiography. The findings should enable the clinician to decide the time, type (gelfoam/platinum coil etc.) and the chances of successful angioembolisation.

We noted that the MDCT angiogram detected other findings such as residual stones, pelvicalyceal system clots, and perinephric hematomas and collections. MDCT angiograms also reveal the venous anatomy and can help to assess the status of the opposite kidney.

Complications following percutaneous vascular access include hemorrhage, arterial obstruction, pseudoaneurysm and AV fistula at the site of access. In addition there is a risk of non-target organ embolisation as well as contrast induced nephropathy (CIN).

The MDCT angiography is also limited by the fact that it cannot be used in the presence of renal insufficiency, as there remains a risk of contrast-induced nephropathy ^10^. MDCT angiography is also contraindicated in patients having sensitivity to contrast agents. The limitations of our study include a small sample size. As MDCT and CA findings were interpreted by different individuals in this study there may have been observer’s variations in the findings. The real value of MDCT is in its potential to identify patients who do not have an identifiable lesion, thus avoiding the need for a CA. This aspect was beyond the role of the present study.

## Conclusion

MDCT is rapid, reproducible and noninvasive. MDCT angiography performed in the setting of post PCNL bleeding provides an accurate assessment of the site and nature of bleeding. MDCT angiography findings correlated with the CA findings in all patients in the present study. These findings need to be further validated in larger multicenter comparative studies.

## References

[1] Martin X Murat FJ Feitosa LC: Severe bleeding after nephrolithotomy: results of hyperselective embolization. *Eur Urol.* 2000;37(2):136–9 10.1159/000020129 10705189

[2] Muller DW Shamir KJ Ellis SG: Peripheral vascular complications after conventional and complex percutaneous coronary interventional procedures. *Am J Cardiol.* 1992;69(1):63–8 10.1016/0002-9149(92)90677-Q 1729869

[3] Anthony S Milburn S Uberoi R: Multi-detecto CT: review of its use in acute GI haemorrhage. *Clin Radiol.* 2007;62(10):938–49 10.1016/j.crad.2007.02.019 17765458

[4] Laing CJ Tobias T Rosenblum DI: Acute Gastrointestinal bleeding: emerging role of Multidetector CT angiography and review of current imaging techniques. *Radiographics.* 2007;27(4):1055–70 17620467 10.1148/rg.274065095

[5] Fishman EK Ney DR Heath DG: Volume rendering versus maximum intensity projection in CT angiography: what works best, when, and why. *Radiographics.* 2006;26(3):905–22 16702462 10.1148/rg.263055186

[6] Rastinehad AR Andonian S Smith AD: Management of hemorrhagic complications associated with percutaneous nephrolithotomy. *J Endourol.* 2009;23(10):1763–1767 10.1089/end.2009.1548 19747040

[7] Richstone L Reggio E Ost MC: First Prize (tie): Hemorrhage following percutaneous renal surgery characterization of angiographic findings. *J Endourol.* 2008;22(6):1129–1135 10.1089/end.2008.0061 18498232

[8] Sivanandam SE Mathew G Bhat SH: Emerging role of multi-detector computed tomography in the diagnosis of hematuria following percutaneous nephrolithotomy: A case scenario. *Indian J Urol.* 2009;25(3):392–394 10.4103/0970-1591.56176 19881138 PMC2779967

[9] Jain V Ganpule A Vyas J: Management of non-neoplastic renal hemorrhage by transarterial embolization. *Urology.* 2009;74(3):522–6 10.1016/j.urology.2008.11.062 19589577

[10] Moos SI van Vemde DN Stoker J: Contrast induced nephropathy in patients undergoing intravenous (IV) contrast enhanced computed tomography (CECT) and the relationship with risk factors: a meta-analysis. *Eur J Radiol.* 2013;82(9):e387–99 10.1016/j.ejrad.2013.04.029 23711425

