# Supplementary material for: Role of multi-detector computed tomography (MDCT) in management of post percutaneous nephrolithotomy (PCNL) bleeding
Source: F1000Res. 2013 Nov 22;2:253. [Version 1] doi: 10.12688/f1000research.2-253.v1 (PMC3968895; doi:10.12688/f1000research.2-253.v1)
Supplement: Peer review history

# Peer review: Referee response for version 1

**Published:** 2014-01-06

**Authors:** Christian Bach

**Affiliations:** 1 Department of Urology, Southmead Hospital, North Bristol NHS Trust, Bristol, UK

The authors present a highly interesting and very well written study evaluating the role of MDCT angiography for bleeding post PCNL, based on a case series. This paper is clearly worth publishing and excellent as it stands. I have solely some suggestions:

- The title should point out that a case series is presented.
   
- All patients had bleeding post PCNL – what exactly were the parameters which triggered the angioembolization? Were there patients where MDCT angiography had been performed, and as a results NO embolization had been performed?
   
- Otherwise, I join in with the comments of the first reviewer.

I have read this submission. I believe that I have an appropriate level of expertise to confirm that it is of an acceptable scientific standard.

# Peer review: Referee response for version 1

**Published:** 2013-12-13

**Authors:** Noor Buchholz, Stefanos Kachrilas

**Affiliations:** 1 Urology Department, Endourology and Stones Services, Barts and The London NHS Trust, London, UK

I have a few minor comments:

1. The very small number of patients does not allow for the extraction of safe conclusions.
   
2. The embolisation should be conducted by an interventional radiologist and not an interventional nephrologist, please provide an explanation as to why this occured.
   
3. The literature results from conventional angiography that the authors provide are excellent, however references regarding the success rates of conventional angiography success rates in post pcnl embolisation are also needed.

We have read this submission. We believe that we have an appropriate level of expertise to confirm that it is of an acceptable scientific standard.
